# A Virtual Reality Game to Assess OCD Symptoms

**DOI:** 10.3389/fpsyt.2020.550165

**Published:** 2021-01-22

**Authors:** Martine J. van Bennekom, Pelle P. de Koning, Martin J. Gevonden, M. Soemiati Kasanmoentalib, Damiaan Denys

**Affiliations:** ^1^Department of Psychiatry, University of Amsterdam, Amsterdam University Medical Centers, Amsterdam, Netherlands; ^2^Department of Biological Psychology, Vrije Universiteit Amsterdam, Amsterdam University Medical Centers, Amsterdam, Netherlands

**Keywords:** virtual reality, game, obsessive-compulsive disorder, assessment, diagnosis

## Abstract

**Background:** Symptoms of obsessive-compulsive disorder (OCD) are often underreported by patients and mainly triggered in the patients private domain, making it harder for clinicians to recognize OCD. Virtual reality (VR) can be used to assess OCD symptoms in the clinician's office. We developed a VR game in order to provoke subjective and physiological OCD symptoms. We hypothesize that (1) the VR game provokes more OCD symptoms in patients compared to healthy controls, (2) performing virtual compulsions leads to a reduction in emotional responses in OCD patients and that (3) the severity of VR game provoked symptoms correlates with severity of OCD symptoms.

**Methods:** Participants played the VR game on a laptop while physiological measures were recorded simultaneously. We measured emotional responses, virtual compulsions and physiological arousal in response to our VR game in 26 OCD patients and 26 healthy controls. We determined correlations between emotional responses, virtual compulsions and OCD severity.

**Results:** We found higher levels of VR-provoked anxiety (U = 179.5, *p* = 0.004) and virtual compulsions in OCD patients compared to healthy controls (*p* = 0.001). There was a significant reduction in emotional responses after performing virtual compulsions in the OCD patients. The emotional responses and virtual compulsions did not correlate significantly with Y-BOCS scores. A baseline difference between patients and healthy controls was found in heart rate variability (HRV), but no significant change in HRV, heartrate and skin conductance was found during the VR game

**Conclusions:** Our study clearly shows our OCD VR game is capable of provoking more anxiety and virtual compulsions in patients with OCD compared to healthy controls. Providing a direct patient-rated measurement in the clinicians room, the VR game could help in assessing core OCD symptoms and recognizing OCD.

**Clinical Trial Registry Number:** Netherlands Trial Register NTR5935.

## Introduction

Obsessive-compulsive disorder (OCD) is a heterogeneous and persistent psychiatric disorder with a lifetime prevalence of 2.3% (1). OCD is characterized by obsessions on varying dimensions including pathological doubt, contamination fear, aggressive or sexual thoughts, and compulsions which may include checking or cleaning compulsions and mental or ordering rituals. The obsessions and compulsions cause abundant anxiety or tension, and lead to impairment in every aspect of daily life functioning (2).

OCD is often poorly recognized, leading to a significant delay between onset of OCD symptoms and initiation of effective treatment; in a study conducted by Hollander et al., a 17.2 year delay was found (3) Patients can have difficulties expressing their symptoms due to shame (4). Moreover, their symptoms are most pronounced in private areas like their home or work environment; they generally do not experience complaints in the safe environment of the clinicians office. This impedes a direct observation of the core symptoms of obsessions, emotional responses, and compulsions by the clinician, making it harder to recognize and diagnose OCD, especially for relatively unexperienced clinicians in primary care. An assessment of OCD is thus based on retrospective and possibly incomplete information from the patient and subjective interpretation of the clinician, increasing the risk of recall and interviewer bias, respectively (5). A direct observation and measurement of the core symptomatology of OCD in the clinical office would result in a more objective and comprehensive assessment of OCD.

Virtual reality (VR) offers new possibilities to achieve this goal. VR is defined as a computerized environment in which a person can be immersed to navigate and interact, designed to simulate lifelike situations (6). It can be used to provoke clinical symptoms in the safe and controlled environment of a clinicians office. Moreover, in VR, patients can directly rate their symptoms, without the intervention of a clinician, which could decrease the risk of underreporting due to shame. Numerous research groups already have investigated the potential of VR to measure symptoms of psychiatric disorders. In most of these studies, a virtual environment was able to simultaneously provoke and measure psychiatric symptoms [for a review, see (7)].

Three studies have examined VR environments to provoke OCD symptoms. Kim et al. studied subjective anxiety and checking compulsions in a virtual house and office. They found higher levels of anxiety and checking compulsions in response to the VR environments in OCD patients compared to healthy controls (8, 9). In a small pilot study with four OCD patients, Belloch et al. found increasing levels of anxiety and disgust on a Visual Analog Scale (VAS) in virtual settings with increasing contamination (10). Likewise, LaForest et al. provoked anxiety in OCD patients with contamination fear in a virtual contaminated toilet. They reported an increase in anxiety and heartrate of OCD patients (11). Kim also found a correlation between Yale-Brown Obsessive Compulsive Scale (Y-BOCS, 12) scores and time spent on checking compulsions in VR in the OCD patient group, but no correlation between Y-BOCS scores and provoked anxiety (9). All study groups made use of animated VR on one single OCD subtype.

The use of video VR involving multiple OCD subtypes to provoke and assess OCD has never been investigated before. In this study we evaluated a video VR game to provoke and assess core OCD symptoms. The VR game includes the OCD subtypes contamination fear and cleaning compulsions, doubt and checking compulsions and symmetry obsessions and ordering compulsions. In a pilot study including 8 patients and 8 healthy controls, we found OCD patients performed more compulsions during the VR game compared to healthy controls. The OCD patients also experienced higher intensity of emotional responses, including anxiety and uncertainty, although the sample size was too small to proof a significant difference. Furthermore, the VR game was tolerated well by OCD patients, it did not cause adverse events (12).

If our VR game is capable of provoking and measuring core symptoms in OCD patients, it could serve as an additional diagnostic tool to help clinicians recognize OCD and speed up initiation of treatment. The purpose of this study is to see whether our video VR game is indeed capable of provoking and assessing OCD symptoms in patients compared to healthy controls. Following from our pilot study, this study includes a larger sample size as well as the addition of physiological arousal measures. We hypothesized that our video VR game (1) leads to a larger increase of emotional responses (anxiety, tension, uncertainty, urge to control), compulsions and physiological arousal in OCD patients as opposed to healthy controls, (2) performing virtual compulsions leads to a reduction in emotional responses in OCD patients and (3) that the severity of OCD symptoms in the VR game correlates with the symptom severity in OCD patients as measured by traditional diagnostic tools.

## Materials and Methods

### Participants

Patients were recruited at the Psychiatric Department of the Academic Medical Center of the University of Amsterdam and Dutch OCD websites. Recruitment took place from June 2014 to December 2017. All included patients had a primary diagnosis of OCD as determined by a psychiatrist according to Diagnostic and Statistical Manual of Mental Disorders-IV (DSM-IV) criteria using the Structured Clinical Interview for DSM-IV Axis I disorders (13). Healthy controls were recruited through advertisements and were included if they were free of current mental disorders as determined by a psychiatrist and validated with the MINI-International Neuropsychiatric Interview (MINI) (14). We excluded subjects with a history of severe neurological or cardiovascular disorders, psychotic disorder, bipolar disorder, intellectual disability, and alcohol or substance abuse during the last 6 months. Enrolled subjects were not allowed to use alcohol or recreational drugs use 24 h prior to investigation. We included 26 OCD patients and 26 healthy controls in an allocated age range of 18–65 years. The magnitude of the sample was based on the ability to detect large group differences (d = 0.8) with 80% power at 5% alpha. Demographic data of the population are shown in Table 1. The study was approved by the Medical Ethics Committee of the Academic Medical Center of the University of Amsterdam and all participants gave written informed consent before enrollment.

**Table 1:** Demographic data of OCD patients and healthy controls.

	**Patients (*****n*** **= 26)**		**Controls (*****n*** **= 26)**		***P*-value**
Age (SD), y	36.5	(11.7)	34.6	(15.9)	0.27
Male sex (%)	12	(46)	12	(46)	1.00
Dutch nationality (%)	24	(92)	26	(100)	0.49
Tertiary education (%)	12	(46)	13	(50)	1.00

### Procedure

At the test day, trained psychiatrists first administered questionnaires, including a demographic questionnaire, the Y-BOCS, Hamilton Rating Scale for Depression [HRSD, (15)], the Igroup presence questionnaire [IPQ, (16)] and the MINI. Presence is defined as the sense of subjectively being in the virtual environment (17). The OCD subtypes were determined with the Y-BOCS symptom checklist (18) and expert opinion of the psychiatrists.

Participants were connected to the physiological measurement device VU University Ambulatory Monitoring System [VU-AMS, (19)] and watched a calm movie with nature scenes with a duration of 5 min on the Lenovo laptop screen to conduct a baseline measurement of emotional responses and physiological arousal. Afterwards, they performed the VR game on the same laptop whilst wearing headphones and whilst physiological recording using the VU-AMS continued. The laptop was connected to a separate screen on which the researchers logged each item in the game for later synchronization with the physiological recording.

### Materials

#### VR Game

**Figure 1:**
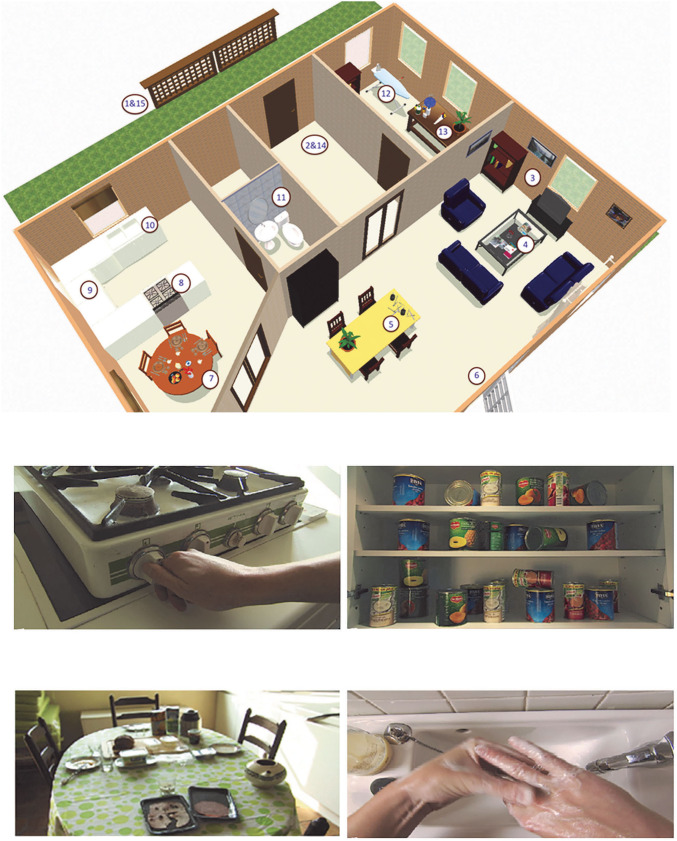
**(A)** 3D-map of house indicating OCD-related items (figure reprinted with permission from Cyberpsychology, behavior & social networking, publisher: Mary Ann Liebert, Inc., New Rochelle, NY) 1, locking gate start; 2, locking front door start; 3, switching off television; 4, extinguishing candle; 5, organizing pencils; 6, closing window; 7, cleaning breakfast table; 8, putting off gas; 9, organizing cans; 10, cleaning sink; 11, hand washing after toilet; 12, switching off flat iron; 13, organizing hazardous substances; 14, locking front door end; and 15, locking gate end. **(B)** Screenshots from the VR game (figure reprinted with permission from Cyberpsychology, behavior & social networking, publisher: Mary Ann Liebert, Inc., New Rochelle, NY).

The set-up of the VR game is described and illustrated in detail in our pilot study (12). A schematic outline and screenshots from the VR game are shown in Figures 1A,B (reprinted with permission). In summary, it concerned a first-person perspective game based on video images where the participant walked through a house with 15 OCD-related items like burning gas, a dirty sink, and messy tables. The items represent the contamination/cleaning, doubt/checking and symmetry/ordering subtypes of OCD. Participants were confronted with all these items in a preset order. After confrontation, participants were asked if they wanted to intervene and subsequently check an item, or if they wanted to dismiss and proceed to the next item in the house. If they chose to intervene and subsequently check, a first-person video was shown of the intervention or checking behavior. Afterwards they were asked if they wanted to repeat the check, they could repeat the checks as often as desired. After each confrontation, intervention and check in the game, participants had to rate emotional responses on a VAS scale. The VR-game recorded both the scores on emotional responses, and the amount of virtual checks performed.

#### Virtual Measures

At baseline and during the VR game, participants had to rate emotional responses including anxiety, tension, uncertainty, and urge to control on a digital VAS, range 0–10. The ratings in the VR game appeared at confrontation with an item, after intervening on an item and after checking an item. The amount of checks during each item was tracked automatically and qualified as compulsions. At each item where compulsions were performed, the change in VAS scores was calculated using the VAS score at confrontation minus the VAS score after the last compulsion.

#### Physiological Measures

We used heartrate (HR), heart rate variability (HRV), and skin conductance level (SCL) as peripheral parameters of physiological arousal. These parameters were measured using the VU-AMS, a device designed to continuously measure autonomic nervous system activity by means of seven impedance cardiogram and electrocardiogram electrodes attached to the chest and back as well as skin conductance electrodes wrapped around the index and middle finger. Respiratory sinus arrhythmia (RSA), the HRV measure used in this study, is the variation in heart rate coupled to respiration and considered to reflect parasympathetic activity specifically (20). The physiological data were recorded during the baseline movie and for the entire duration of the VR game. The data were processed using the accompanying data analysis and management software (VU-DAMS, v4.0). We created labels of 30 s around confrontation with the OCD-related items in the VR game. HR and SCL reactivity was determined by subtracting the baseline value from the mean value in each label. To determine HRV, we created extended labels clustering two or three successive OCD-related items in the VR game to ensure sufficient breathing cycles within each label, and used the RSA-0 value in milliseconds.

### Data Processing and Statistical Analysis

To assess overall VR game effects the virtual and physiological data were reduced by averaging the values for the 15 OCD-related items. The resulting mean values and clinical data were not normally distributed. We used Fisher's Exact test for comparison of categorical data (sex, ethnicity, schooling, questionnaire outcomes, number of compulsions). We used the Mann-Whitney U test for comparison of continuous data (age, emotional responses, physiological data). Regarding the four emotional responses, a Bonferroni correction was performed to correct for multiple testing. A one-sample Wilcoxon Signed Rank Test was used to assess the effect of the VR game on emotional responses and physiological data, and to assess the reduction in emotional responses after performing compulsions in patients. To calculate correlations, a Spearman's Rho was used. Alpha was set at 0.05. All statistical tests were computed with SPSS (IBM SPSS Statistics for Windows, version 24).

## Results

### Participants

Demographic data did not differ significantly between OCD patients and healthy controls (Table 1). Clinical data including scores on the obtained questionnaires, use of medication and OCD subtypes are shown in Table 2. Almost all patients had symptoms from multiple OCD subtypes, Table 2 shows the dominant OCD subtype. The most prevalent dominant OCD subtypes included doubt/checking (34%) and contamination/cleaning (31%). Of the OCD patients who used antidepressants, three used a tricyclic antidepressant and 18 used a selective serotonin (and/or noradrenalin) reuptake inhibitor. Five patients used an antipsychotic in addition to their antidepressant. Seven OCD patients suffered from a comorbid depression, four patients suffered from a comorbid anxiety disorder. All three components of the Igroup presence questionnaire, which was obtained after the VR game, did not differ between OCD patients and healthy controls (HC); mean spatial presence patients 3.19 (SD 0.87), HC 3.16 (SD 0.94), *p* = 0.79, mean involvement patients 3.01 (SD 1.62), HC 3.05 (SD 1.57), *p* = 0.91, mean realism patients 3.02 (SD 1.04), HC 2.75 (SD 0.96), *p* = 0.19.

**Table 2:** Clinical data of OCD patients and healthy controls.

	**Patients (*****n*** **= 26)**		**Controls (*****n*** **= 26)**		***P*-value**
	***n***		***n***		
**Y-BOCS score (%)**					
Mild (8–15)	7	(26.9)	NA		
Moderate (16–23)	12	(46.2)			
Severe (> 23)	7	(26.9)			
**OCD subtype (%)**					
Contamination	8	(30.8)	NA		
Symmetry	6	(23.1)			
Doubt	9	(34.6)			
Other	3	(11.5)			
**HAM-D score (%)**					
Normal (0–7)	8	(30.8)	26	(100)	0.00
Mild (8–13)	11	(42.3)			
Moderate-severe (>14)	7	(26.9)			
**HAM-A score (%)**					
Mild (0–17)	20	(76.9)	26	(100)	0.023
Moderate-severe (18–30)	6	(23.1)			
**Medication (%)**					
None	5	(19.2)	NA		
Antidepressant	21	(80.8)			

*Y-BOCS, Yale-Brown Obsessive–Compulsive scale; HAM-D, Hamilton Depression rating scale; HAM-A, Hamilton Anxiety rating scale*.

### Virtual Measures

In order to compare the increase in emotional responses between patients and healthy controls, we first tested whether performing the VR game led to an increase in emotional responses compared to baseline in both groups. The baseline scores on all four emotions are shown in Table 3A and were significantly higher for OCD patients than for healthy controls. The provoked emotional responses (mean ΔVAS scores), defined as the mean VAS score at confrontation over all 15 OCD-related items in the VR game minus the VAS scores at baseline can be seen in Table 3B. Both in the patient and healthy control group, compared to baseline values, performing the VR game increased significantly anxiety, tension, and urge to control but not uncertainty.

**Table 3A:** Emotional responses at baseline.

	**Patients (*****n*** **= 26)**		**Controls (*****n*** **= 26)**		**U-value**	***P*-value**
	**VAS**		**VAS**			
Anxiety (SE)	0.88	(0.22)	0.04	(0.04)	179	<0.001
Tension (SE)	1.77	(0.34)	0.27	(0.16)	111.5	<0.001
Uncertainty (SE)	1.73	(0.45)	0.23	(0.16)	163	<0.001
Urge to control (SE)	1.42	(0.49)	0.00	(0.00)	208	0.001

*SE; standard error of the mean, VAS; visual analog scale*.

**Table 3B:** Provoked emotional responses.

	**Patients (*****n*** **= 26)**		***P*-value**	**Controls (*****n*** **= 26)**		***P*-value**
	**Δ VAS**			**Δ VAS**		
Δ Anxiety (SE)	1.73	(0.45)	0.001	0.24	(0.1)	0.005
Δ Tension (SE)	1.26	(0.47)	0.007	0.44	(0.18)	0.001
Δ Uncertainty (SE)	0.79	(0.46)	0.097	0.11	(0.19)	0.041
Δ Urge to control (SE)	1.63	(0.66)	0.008	0.96	(0.25)	<0.001

*SE, standard error of the mean; VAS, visual analog scale*.

**Figure 2:**
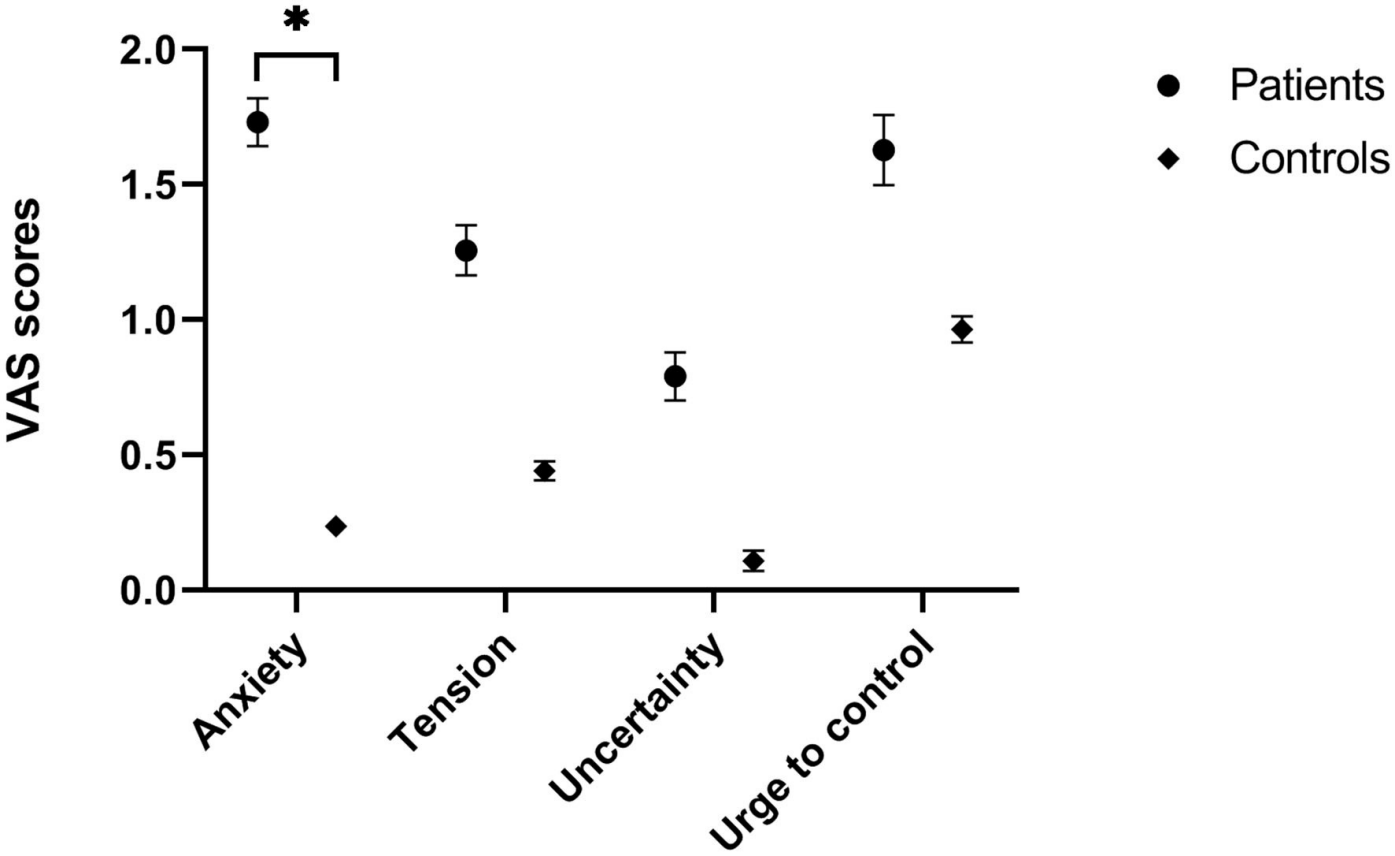
Emotional responses (mean and SE) compared to baseline during VR game.

As can be seen in Figure 2, the increase in anxiety was significantly higher for OCD patients than for healthy controls (U = 179.5, *p* = 0.004). Although both tension (U = 245.5, *p* = 0.09), uncertainty (U = 268, *p* = 0.197) and urge to control (U = 276.5, *p* = 0.26) were also higher for patients, these differences did not reach significance.

We also compared the amount of performed compulsions between the two groups. OCD patients performed significantly more compulsions during the VR game compared to healthy controls (mean patients 7.38, SE 1.63 mean HC 1.50, SE 0.28, *p* = 0.001). Of all the patients who performed more than 10 compulsions, two performed over 20 compulsions throughout the game. There were no healthy controls who performed more than 5 compulsions throughout the game.

Furthermore, in OCD patients, we investigated whether performing compulsions led to a decrease in provoked emotions. OCD patients experienced both a significant reduction in anxiety (mean 1.04, SE 0.28, *p* = 0.001), tension (mean 1.14, SE 0.29, *p* = 0.001), and urge to control (mean 1.40, SE 0.43, *p* = 0.003). We did not find a significant difference in the reduction of uncertainty (mean 0.65, SE 0.27, *p* = 0.013).

### Physiological Measures

Due to technical problems, we were unable to obtain reliable physiological measures for one patient and three healthy controls. Therefore, the analyses for physiology pertain to 25 OCD patients and 23 healthy controls. We first tested whether performing the VR game led to a change in physiological measures (reactivity) compared to baseline in both groups. The baseline scores on physiological measures are shown in Table 4A; baseline HR and SCL did not significantly differ between patients and healthy controls. However, OCD patients had a significantly lower HRV at baseline compared to healthy controls, while the respiration rate (RR) at baseline did not differ significantly. For both groups, the mean reactivity of HR, SCL, and HRV at confrontation with the 15 OCD-related items during the VR game compared to baseline can be seen in Table 4B.

**Table 4A:** Physiological measures at baseline.

	**Patients (*****n*** **= 25)**		**Controls (*****n*** **= 23)**		**U-value**	***P*-value**
HR (SE)	69.0	(1.87)	69.1	(1.89)	276	0.812
SCL (SE)	6.07	(0.57)	8.00	(0.81)	205	0.089
HRV (SE)	46.87	(7.92)	66.74	(7.62)	174	0.019
RR (SE)	15.69	(0.47)	14.93	(0.46)	219	0.157

*HR, heartrate (bpm); SCL, skin conductance level (μS); HRV, heart rate variability (ms); RR, respiration rate (bpm)*.

**Table 4B:** Physiological reactivity.

	**Patients (*****n*** **= 25)**		***P*-value**	**Controls (*****n*** **= 23)**		***P*-value**
**Δ Physiology**						
ΔHR (SE)	0.18	(0.55)	0.840	−1.59	(0.58)	0.018
ΔSCL (SE)	0.56	(0.32)	0.166	0.43	(0.38)	0.191
ΔHRV (SE)	−3.30	(2.51)	0.493	−4.16	(2.61)	0.224

*HR, heartrate (bpm); SCL, skin conductance level (μS); HRV, heart rate variability (ms)*.

**Figure 3:**
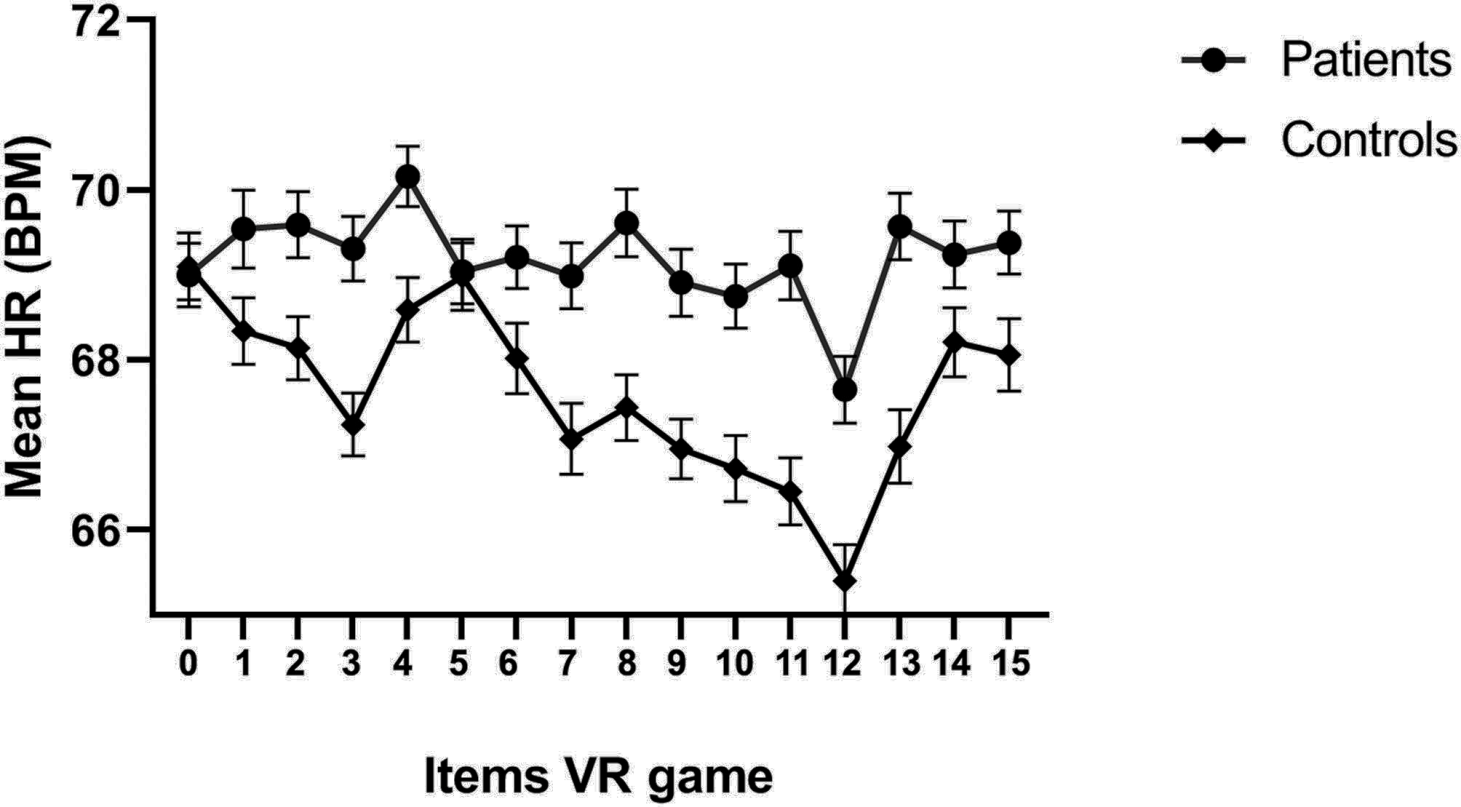
Mean heart rate and SE per item.

Both in the patient and healthy control group the mean scores at confrontation with the OCD-related items in the VR game for SCL and HRV did not significantly differ compared to baseline scores. This was also true for the mean HR in the patient group. However, there was a significant decrease in HR compared to baseline in the healthy control group. To illustrate, Figure 3 shows the mean heart rate during each item of the VR game for each group, where 0 represents the baseline and the subsequent numbers the 15 items of the VR game. For the control group, there is a visibly decreasing trend in heart rate in the course of the game from baseline. Confrontations with a burning candle (item 4), the left on gas-burner (item 8) and hazardous substances (item 13) appear associated with elevations in heart rate in both groups, and toward the end of the game (item 14 and 15, locking of door and gate), the heart rate is elevated again.

We found no significant differences in HR reactivity (U = 193, *p* = 0.051), SCL reactivity (U = 279, *p* = 0.861), and HRV reactivity (U = 271, *p* = 0.733) between patients and healthy controls.

### Correlations

The mean total Y-BOCS score of the OCD patient population was 20.81 (SD 7.04), with a mean of 10.31 (SD 3.48) and 10.50 (SD 3.90) on the obsession and compulsion subscale, respectively. No significant correlations were found between total Y-BOCS scores and mean ΔVAS scores on anxiety (ρ = 0.26, *p* = 0.21), tension (ρ = 0.20, *p* = 0.34), uncertainty (ρ = 0.02, *p* = 0.93 and urge to control (ρ = 0.25, *p* = 0.22) for the OCD patients. Furthermore, no significant correlation was found between total Y-BOCS scores and number of performed compulsions during the game (ρ = 0.27, *p* = 0.189), additionally no significant correlation was found between the compulsion subscale Y-BOCS score and the number of performed compulsions during the game (ρ = 0.31, *p* = 0.119).

## Discussion

In this study, we tested whether our specifically designed video VR game was able to provoke a larger increase in subjective and physiological symptoms in OCD patients as opposed to healthy controls. Firstly, as hypothesized, our data clearly show our VR game was able to trigger both higher levels of anxiety and virtual compulsions in OCD patients compared to controls. This is in line with preliminary results of our pilot study and proofs that the VR game is able to provoke core symptoms of OCD, despite the heterogeneity in OCD subtypes of the patients. Indeed, participating patients often indicated afterwards the items in the VR-game were able to trigger their obsessions and they felt a strong urge to perform virtual compulsions. In contrast, we found no relevant differences in provoked tension, uncertainty and urge to control between patients and controls. OCD patients experienced higher levels of all emotions at baseline, making it more difficult to detect an increase during performance of the VR game. The higher emotional levels at baseline could be caused by distress in anticipation of the VR game, which was new and challenging to them and possibly by the preceding administration of questionnaires.

Secondly, we found a significant reduction in both anxiety, tension and urge to control, but not in uncertainty after performing virtual compulsions in OCD-patients. This mostly supports the cognitive behavioral theory, in which OCD patients perform compulsions to reduce distress associated with obsessions (21). However, the lack of reduction of uncertainty could be more in line with the habit theory of compulsivity, with a shift of compulsions as goal-directed actions toward habitual behavior during the course of OCD (22). In addition, some patients indicated compulsions in the VR game were not performed exactly how they would have performed them in real life which could also explain why they still experienced uncertainty afterwards. However, this still provides the clinician with relevant information in how the compulsions of the specific patients differ from the compulsions as performed in the VR-game.

Thirdly, we found no correlation between provoked emotions during the VR game and the Y-BOCS, which is used as the golden standard to indicate OCD severity. This could be explained by the difference in set-up of both measurements. The Y-BOCS measures the severity of obsessions and compulsions over the preceding days. The VR-game measures current emotional responses to stressful virtual situations and performed virtual compulsions. One could argue the Y-BOCS represents a state measurement and the VR-game a trait measurement. Therefore, in a future study, it would be interesting to investigate a correlation between anxiety provoked by the VR-game and trait anxiety, using for example the trait part of the State-Trait Anxiety Inventory [STAI, (23)].

Our results on provoked emotions and virtual compulsions correspond in large measure to previous studies on VR and OCD. Kim et al. also found higher levels of anxiety, a larger decrease in anxiety after checking and a higher frequency of checking behavior in patients compared to controls in a virtual house and office. Furthermore, they found a positive correlation between Y-BOCS scores and both anxiety and checking time (8, 9). Likewise, Laforest et al. found a higher level of provoked subjective anxiety after immersion in a virtual contaminated toilet in OCD patients of the contamination subtype compared to controls (11). All results are supportive on the use of VR as a method to provoke and assess OCD symptoms, despite the use of various VR display techniques and software with animated VR being used in the studies of Kim et al. (8) and LaForest et al. (11).

Finally, we tested whether our VR game was able to provoke more physiological responses in OCD patients as opposed to healthy controls. We did not find a significant difference in mean heart rate, skin conductance level and heart rate variability during the VR game compared to baseline between the two groups. In the healthy control group, we did find a significant decrease in heartrate during the VR game compared to baseline. Possibly healthy controls are able to calm down more compared to the patients during performance of the VR game after some initial anticipation anxiety. In the OCD patient group, we did not find a significant effect of the VR game as a whole on HR, SCL, and HRV. When looking in detail at the heartrate during the VR game however, there is a clear effect of several items in the VR game on HR. At the end of the VR game, especially OCD patients frequently explained they were afraid to leave the house since they were not given the opportunity to make a final check-up of all items, which could explain the elevated trend in heartrate toward the end of the VR game.

In contrast to our study, Laforest et al. did find a significant increase in heart rate when OCD patients were immersed in the virtual environment, though they only tested OCD patients of the contamination subtype in a correspondent virtual environment (11). In our study, patients with different OCD subtypes were tested in the same environment. Furthermore, they used a cube-like space displaying images (CAVE system) to study their virtual environment, which is more immersive than our virtual environment on a laptop screen. Therefore, we hypothesize it takes a more immersive VR environment which corresponds with the OCD subtype to elicit a detectable physiological arousal.

Interestingly, we did find a lower HRV at baseline in OCD patients relatively to controls, which was not explained by a baseline difference in heartrate or respiration rate. The baseline difference in HRV could be indicative of lower parasympathetic activity and less flexible adaptation to internal or environmental conditions in the OCD patient group (20). Former studies found lower resting state HRV on most anxiety disorders, but not OCD (24, 25). The study of Pittig et al. did find a lower baseline HRV for OCD patients, however this result was significantly influenced by current psychotropic medication use (26). In our study, most OCD patients used psychotropic medication as well and some suffered from a comorbid anxiety or depressive disorder, which could have had an effect on HRV measurements.

Our study has a few limitations. Firstly, even though the VR game represented multiple OCD subtypes, it was not possible to include all subtypes in the game. We did not select OCD patients based on their OCD subtype, which could have moderated our results. In a larger sample, it would be interesting to perform a *post-hoc* analysis on OCD subtype including only the subtype-specific items of the VR game. Secondly, our patient group used different types of psychotropic medication and suffered from different comorbid disorders, which could have influenced the physiological measurements. We deliberately chose a clinically relevant sample of OCD patients to enhance ecological validity, which means we did not exclude patients based on comorbid (non-severe) disorders and the use of medication. Thirdly, we did not measure intra-patient test-retest reliability in this study. Since the VR-game consists of a specific amount of OCD related items, we would expect the responses to be maximal at the first time of performance, due to habituation after repeated presentation. In a future study at our department, OCD patients will perform the VR-game before and after treatment with cognitive behavioral therapy, to be able to determine if the VR-game can be used to assess treatment effects.

All in all, our findings indicate the VR game is able to provoke core symptoms of anxiety and virtual compulsions in OCD patients as opposed to healthy controls. Furthermore, OCD patients experienced a significant reduction in emotional responses after performing virtual compulsions. More studies with larger sample sizes would be necessary to determine threshold values on VR-game measures to distinguish between OCD patients and healthy controls. There was no correlation between provoked emotions or compulsions and Y-BOCS scores. The addition of physiological measures to the VR game so far had limited additional value since no differences were found in provoked heartrate, skin conductance and heart rate variability during the VR game between the patient and control group.

Providing a direct patient rated measurement in the clinicians room, the VR game allows clinicians to assess specific items triggering compulsions, the type and intensity of provoked emotions, and if compulsions provide relief or have to be repeated continuously without a reduction in emotions. This detailed information on core symptomatology could be of help in assessing OCD symptoms and recognizing OCD, especially for less experienced clinicians. In future studies, it would be interesting to investigate if the VR game could identify the dominant OCD subtype in a patient, especially if we would include more OCD subtypes in a new version of the VR-game. Furthermore, a future study to demonstrate if the VR-game can be used to recognize and diagnose OCD more quickly in primary care settings, could be of value in addressing the large gap between initiation of OCD symptoms and start of treatment.

## Data Availability Statement

The raw data supporting the conclusions of this article will be made available by the authors, without undue reservation.

## Ethics Statement

The studies involving human participants were reviewed and approved by Medical Research Ethics Committee, Academic Medical Centre (AMC), Amsterdam. The patients/participants provided their written informed consent to participate in this study.

## Author Contributions

MB, PK, MK, and DD contributed to the conception of this manuscript. MB, MK, and MG collected and interpreted the data for this manuscript. MB drafted this manuscript. All authors discussed the results, critically revised this manuscript, approved the submitted version of this manuscript, and agree to be accountable for all aspects of the work.

## Conflict of Interest

The authors declare that the research was conducted in the absence of any commercial or financial relationships that could be construed as a potential conflict of interest.
